# Virtual Coaching for Rehabilitation: The Participatory Design Experience of the vCare Project

**DOI:** 10.3389/fpubh.2021.748307

**Published:** 2021-12-02

**Authors:** Agnese Seregni, Enrica Tricomi, Peppino Tropea, Rocio Del Pino, Juan Carlos Gómez-Esteban, Inigo Gabilondo, María Díez-Cirarda, Hannes Schlieter, Kai Gand, Massimo Corbo

**Affiliations:** ^1^Department of Neurorehabilitation Sciences, Casa di Cura del Policlinico, Milan, Italy; ^2^Institut für Technische Informatik (ZITI), Heidelberg University, Heidelberg, Germany; ^3^Neurodegenerative Diseases Group, Biocruces Bizkaia Health Research Institute, Barakaldo, Spain; ^4^Department of Neurology, Cruces University Hospital, Barakaldo, Spain; ^5^Ikerbasque: The Basque Foundation for Science, Bilbao, Spain; ^6^Faculty of Business and Economics, Technische Universität Dresden, Dresden, Germany

**Keywords:** user-centered design, virtual coaching, rehabilitation, stroke, Parkinson's disease, usability and user experience

## Abstract

End-user involvement constitutes an essential goal during the development of innovative solution, not only for the evaluation, but also in codesign, following a user-centered strategy. Indeed, it is a great asset of research to base the work in a user-centered approach, because it allows to build a platform that will respond to the real needs of users. The aims of this work are to present the methodology adopted to involve end-users (i.e., neurological patients, healthy elderly, and health professionals) in the evaluation of a novel virtual coaching system based on the personalized clinical pathways and to present the results obtained from these preliminary activities. Specific activities involving end-users were planned along the development phases and are referred to as participatory design. The user experience of participatory design is constituted by the two different phases: the “end-user's perspective” phase where the user involvement in experiential activities is from an observational point of view, whereas the “field study” phase is the direct participation in these activities. Evaluation tools (i.e., scales, questionnaires, and interviews) were planned to assess different aspects of the system. Thirty patients [14 with poststroke condition and 16 with Parkinson's disease (PD)], 13 healthy elderly, and six health professionals were enrolled from two clinical centers during the two phases of participatory design. Results from “end-user's perspective” phase showed globally a positive preliminary perception of the service. Overall, a positive evaluation (i.e., UEQ median score > 1) was obtained for each domain of the scale in both groups of patients and healthy subjects. The evaluation of the vCare system during the “field study” phase was assessed as excellent (>80 points) from the point of view of both patients and health professionals. According to the majority of patients, the rehabilitation service through the solution was reported to be interesting, engaging, entertaining, challenging and useful for improving impaired motor functions, and making patients aware of their cognitive abilities. Once refined and fine-tuned in the aspects highlighted in the this work, the system will be clinically tested at user's home to measure the real impact of the rehabilitative coaching services.

## Introduction

The continuity of care and personalized rehabilitation for people who experienced acute episodes or who are affected by chronic diseases is often interrupted after transitioning from the hospital to the home environment. Home rehabilitation measures require the presence of a coach following the patient along the assigned care plan (1).

Smart solutions based on a virtual coach (VC) can help to provide a personalized home rehabilitation plan according to the patient's condition and habits to enhance the quality of life (QoL) and to empower patients toward a healthy lifestyle. In this scenario, innovative information and communication technologies (ICT) system based on an intelligent avatar, acting as a VC, could offer home rehabilitation programs to elderly subject or to patients with neurological and cardiological issues. The avatar could be displayed on a mobile device or on a smart television to communicate with patients mainly through natural speech communication. The whole architecture could be arranged in a smart digital environment composed of environmental sensors, wearable sensors, and gaming devices.

The main goal of a new generation of VC is to assist and counsel the patient during home rehabilitation activities. Immersive virtual simulations may represent a potentially effective training tool over and above existing methods for training primary care providers (2).

Indeed, several projects have recently focused on VCs development to improve patients' rehabilitation through an intelligent environment, integrating machine learning technologies together with well-elaborated coaching and clinical pathway services (1).

The vCare project, funded under the European Commission (EC) Horizon 2020 call “personalized coaching for well-being and care of people as they age” (SC1-PM15-2017), fits into this context by proposing a new ICT-based concept. The proposed platform encapsulates a set of coaching services for empowering and motivating people, which helps them to proceed with a personalized rehabilitation that complies with age-related physical, cognitive, mental, and social conditions by a VC (3).

The project's final solution will particularly be tailored for patients affected by stroke, Parkinson's disease (PD), and then eventually involving cardiovascular patients. These neurological diseases are especially relevant for elderly people (3).

End-user involvement constituted an essential goal in vCare, not only for the evaluation, but also in codesign, following a user-centered strategy. Senior users, suffering from neurodegenerative and other chronic diseases, at different stages and belonging to different social groups, will assess the suitability of the vCare platform at addressing their health and social issues and improving their QoL.

The aims of this work are (i) to present the methodology adopted to involve end-users (i.e., neurological patients, healthy elderly, and health professionals) in the evaluation of a novel VC system based on personalized clinical pathways; (ii) to present the preliminary results obtained on investigating the first users' feedback. The whole testing and validation process of the vCare solution prototype was designed in a user-centered perspective. Specific activities involving end-users were planned along the development phases and are referred to as participatory design. Users' feedback about the system, which gathered through standardized questionnaires, open discussion, and direct experience of the prototype, is taken into consideration along the development process to tailor specific needs and increase the final acceptance.

This work describes the participatory experience of two different types of neurological patients (i.e., subjects affected by stroke or PD) in the evaluation of an ICT system (vCare system). In particular, the overall user-centered evaluation approach is presented, which takes into account the phases related to end-user's perspective and system usability.

### Use of Technologies: Overview

With regard to the inclusion of technology in the daily lives of patients, it has been seen that patients' priorities may differ from those of careers and professionals, so it is important to take all perspectives into account when developing technological tools and making care plans (4).

Patients identify technology as something useful, which allows them to use it for leisure, to increase their freedom and independence (5). They show interest in using technology, although they are less motivated by constant monitoring at home and are concerned about incorporating light and sound warnings and camera-based technologies into their daily lives (6).

Informal careers find that technology incorporated into their lives and the people they care for daily lives provides them with an increased peace of mind and relief from the burden of care. Similarly, formal careers embrace the fact that technologies can ease the monitoring of people in need of care and possibility of interactions with other stakeholders (5).

Health professionals consider that technologies reduce their workload and allow them to devote more attention to patients that require it. However, they believe that for technology to enrich rather than weaken the patient–physician relationship, medical humanism must be in the center of the design thinking behind emerging technologies and software. Most importantly, considerable effort will be needed to plan how technology improves the quality of human interactions rather than simply focusing on efficiency, both among team members and with patients and their families (7).

Families as informal careers give preference to patient safety rather than autonomy when they are responsible for patients. When patients are under the responsibility of formal caregivers, they give preference to patient autonomy rather than patient safety (8).

### Theoretical Framework

When creating such new technological solutions, acceptance and perceived usability by patients and healthcare providers should be evaluated from the earliest stages of the development and be included in the design process to tailor users' specific needs and to reduce the risk of non-acceptance (9). Such a user-centered approach improves user satisfaction and increases usability and functionality (10).

It is well known that the development of drugs goes through several modifications, including understanding its effects and impact on the user prior to making it public and ensuring adoption. None of these medications is developed entirely based on evidence-based literature or personal experiences. This is similar to what user-centered design (UCD) aims to achieve, but with technology solutions (11).

The concept of UCD describes a design and a development process in which the influence of the end-user is considered. UCD is a multidisciplinary and an iterative design process that involves, actively engaging, the users and incorporates their feedback to ensure that tools are developed with a full understanding of their needs and requirements (12, 13).

User-centered design characterizes an iterative design process where the feedback of the user is integrated throughout the whole developing process (13). To take into account different opinions and a broader knowledge, multidisciplinary teams (e.g., clinicians, physiotherapists, and caregivers) are included as well (14). To be able to collect users' feedback, several methods from social sciences were implemented during the development of the solution. These methods include: semi-structured interviews, focus groups, surveys, questionnaires, and clinical evaluations. The added value of using a user-centered approach is to be able to reach a high usability score and to increase users' acceptance of the technology. Being a part of the creation process, users are fully integrated in the definition of the solution. In a user-centered perspective, by focusing on the user throughout the design, development, implementation, and validation of a product, process, or workflow, it is possible to increase both end-user performance and satisfaction.

In general, innovative technological solutions should be developed considering the needs of end-users in a real-life context. Indeed, the UCD approach not only involves the analysis of real demands of end-users, but also the test of the validity of developed solutions or products with regard to user behavior in real scenario (15). To make an effective impact in applications in digital health for rehabilitative purposes, for example, by means of VCs, the acceptance and effectiveness of such solutions are crucial (16).

## Materials and Methods

### Participants

A group of neurological patients, a group of healthy subjects, and a group of health professionals were enrolled from two clinical centers according to the two targeted diseases (i.e., stroke and PD).

Subjects who suffered from an acute stroke event were enrolled among patients of Casa di Cura del Policlinico (CCP), a rehabilitation hospital in Milan (Italy). Patients with PD were enrolled by the Neurology department at Cruces University Hospital and Biocruces Bizkaia Health Research Institute (Osakidetza, OSA). In addition, groups of healthy elderly were enrolled in each site.

The inclusion and exclusion criteria for each clinical center and pathology are reported in Table 1.

**Table 1:** Eligibility criteria for participant's recruitment.

**Group**	**Inclusion criteria**	**Exclusion criteria**
Stroke	i. Age > 65 years; i. Experience of acute stroke event; iii. Ability and acceptance to watch a demonstration video and to proceed with the interview afterwards.	i. Presence of cognitive impairment, other chronic diseases, or psychiatric problems.
Parkinson's disease	i. Parkinson's disease diagnosis according to established clinical criteria (Brain Bank of London) with an index of Hoehn and Yahr between one and three; ii. Ability and acceptance to watch a demonstration video and to proceed with the interview afterwards.	i. Presence of a typical Parkinsonism; ii. Presence of cognitive impairment, other chronic diseases, or psychiatric problems.

Regarding healthy controls, they were recruited taking into account the following exclusion criteria: the presence of cognitive impairments (according to the clinical assessment) that interfere with the ability to willingly understand and give informed consent and age <18 years.

Health professionals were enrolled according to the following eligibility criteria: (i) be qualified and work in a medical or social area specializing in the proper targeted disease; (ii) be part of the clinical team of one of the patients included in the work; (iii) willing to participate in the work; and (iv) able and willing to provide informed consent.

Each participant signed the informed consent forms before the beginning of evaluations and all research procedures were in accordance with the Declaration of Helsinki. Ethics Committees of involved centers reviewed and approved the protocol.

### Overall User-Centered Evaluation Approach of the vCare System

#### Overview

**Figure 1:**
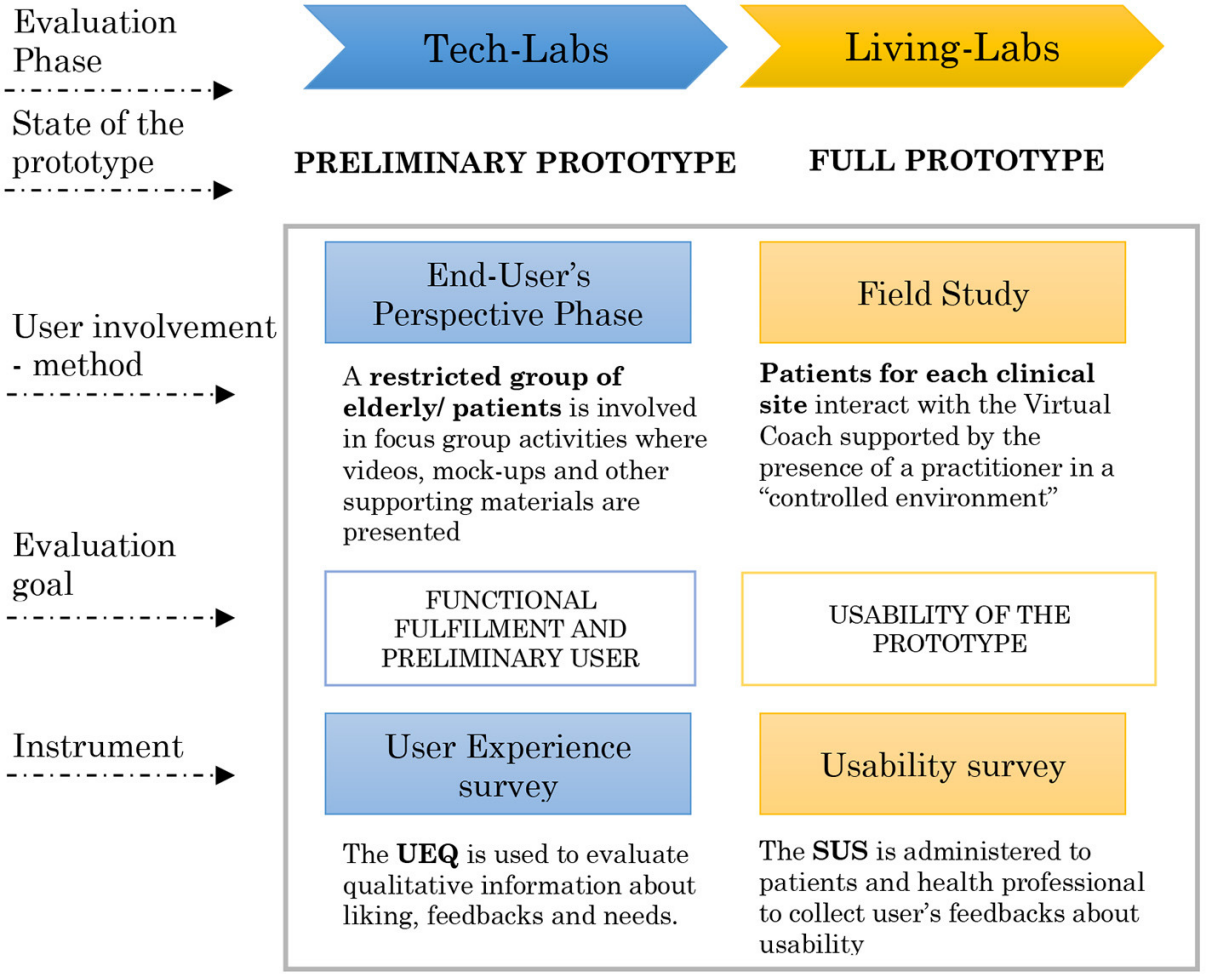
Participatory design concept in the vCare project along the phases of system evaluation.

The vCare prototype is under development in a highly multidisciplinary context, involving physicians (neurologist and cardiologist), physiotherapists, neuropsychologists, bioengineers, and ICT developers. The whole evaluation methodology of the system has been designed as an incremental testing approach composed of three different phases according to our previous work (3) as follows: (1) The Tech Labs (TL) phase, (2) The Living Labs (LL) phase, and (3) The Pilot Tests (PT) phase. The three phases aim to evaluate system functionalities, usability, and acceptability, respectively. Along these phases, the system is tested incrementally in relation to the availability of functional requirements provided by the vCare architecture, integration among different architectural layers, and components. An overview of the phases for vCare testing and validation is presented graphically in Figure 1 (9).

User involvement along the process is referred to as “participatory design”, which is considered the core of vCare evaluation approach. Participatory design is meant to provide valuable suggestions for the deployment process in a user-centered perspective.

Indeed, alongside the evaluation process of the vCare system, particular importance is given to end-users' opinion (i.e., patients and clinical staff). Actually, the user experience of participatory design is constituted by two different phases: the first one is the user involvement in some experiential activities from an observational point of view (end*-*user's perspective phase), whereas the second is the direct participation in these activities (field study phase). Both the evaluation tools (i.e., scales, questionnaires, and interviews) and technical testing were planned to assess different aspects of the system. In fact, the *end-user's perspective* phase and the *field study* phase were the part of TL and LL (Figure 1), respectively, and reported in this work. The longest and final validation of the vCare system, PT phase, will be performed at user's home, and it is not included in this manuscript.

#### The *End-User's Perspective* Phase

In parallel to functional tests (in TL phase), a group of patients and a group of healthy subjects were enrolled to give a preliminary evaluation of the proposed system. At this stage, end-users could not interact with the system, but they acknowledged its features and functionalities through a demonstration video.

This phase (i.e., end-user's perspective) started with a first colloquial interaction (phone call or meeting) with a general presentation to the participants of the burden of neurological disease in Europe and in specific country. The general aim of the vCare project was clarified by the investigator, and further details regarding the end-user involvement in the cocreation process of vCare services were presented. vCare services and functionalities, and also the foreseen interactions with the subjects, were depicted in a video footage meant as a presentation of the system to end-users. Demonstration videos were produced in each clinical center and in their own official language (Italian and Spanish) for the targeted pathologies (https://vcare-project.eu/). Then, vCare concept movie was presented (or shared using online-sharing service) to the enrolled subjects. The CCP center shared the demonstration video through an e-mail sent directly to the subject or to his/her caregiver. Afterwards, each involved subject was asked to watch the vCare storytelling video on their own through personal devices, whereas OSA center showed their demonstration video in a physical meeting with the participants.

After watching the video, an interview was carried out and structured on a two-fold level: user experience questionnaire (UEQ) administration and qualitative semi-structured interview. The interviewer could support the subject with the compilation of UEQ questionnaire items only in case of difficulties, avoiding any bias action. After administering the questionnaire, the interviewer identified (by the items score) the main critical issues highlighted by respondents, and he/she proceeded with a semi-structured interview with qualitative output to investigate in depth the reasons of the most critical issues.

#### The *Field Study* Phase

In the field study phase (during LL), the system prototypes were tested, for the first time, directly by a group of patients in a ‘controlled environment', where patients behaved like at their own homes while staying in the clinic supervised by members of the clinical staff. Specific home-like areas (namely, living labs) were set up into two clinical centers [CCP (Italy) and OSA (Spain)].

Subjects experienced 2-week long rehabilitation treatments autonomously under the supervision of clinicians. Both patients and health professionals interacted directly with the system. Specifically, according to patient's clinical status and clinicians' indications, the vCare system proposed personalized rehabilitation treatments providing a suite of motor and cognitive serious games in a virtual reality (VR) environment. During the motor rehabilitation sessions, a 3D-depth camera is used to recognize the user's movements, which were displayed in the VR environment through a monitor (40-inch-wide screen) in real time. For cognitive rehabilitation activities, the patient interacts instead with a tablet (10-inch). In both activities (i.e., motor and cognitive), the patient is encouraged to reach specific goals in relation to his/her performance. Goals are determined by the system according to characteristic rules; in some cases, distractor was presented to increase the difficulty level of the games.

For both the activities, the patients joined the serious games sessions scheduled, whereas the health professionals assigned a customized serious game rehabilitation plan and performed patient training, support, and supervision during the session. The activities are grouped into packages of serious games with a precise rehabilitation objective; each game package is composed of a number of predefined games addressing a specific clinical/functional aim. The packages and their rehabilitation purposes are reported in Table 2.

**Table 2:** Rehabilitation activities and related clinical/functional purposes.

**Rehabilitative domain**	**Activities**	**Rehabilitation purposes**
Motor	Mobility	To increase joints mobility functions
	Strength	To increase muscular strength against gravity
	Coordination	To improve learning and execution of motor patterns
	Dexterity	To increase grasp, grip, pinch and manipulation functions
	Speed	To accelerate moves
	Motor control	To improve accuracy of motion
	Postural control	To adapt postural organization to different motor transitions
	Balance	To improve stability of center of mass
	Endurance	To train exertion
	Rhythm	To improve smoothness of motion
Cognitive	Attention training	To stimulate attentional skills (e.g., sustained, selective, and shifting)
	Executive functions training	To stimulate several aspects of executive functions (e.g., planning and monitoring, abstraction and categorization)

The usability of the platform user interface and also the subjects' satisfaction with the services were assessed by patients at the end of the intervention through the system usability scale (SUS) (17). Motor and cognitive services were assessed separately. A semi-structured interview was carried out following specific items to investigate general and specific patients' opinions, regarding both motor and cognitive activities experiences. Additionally, SUS questionnaire was filled out by healthcare professionals, who also evaluated the process of patient's characterization and rehabilitation plan's definition inside the vCare platform dedicated to them.

### Outcome Measures and Data Processing

#### User Experience Questionnaire

The UEQ (18) was administered to measure classical usability aspects and user experience aspects. It is a questionnaire composed of 26 items built as pairs of contrasting attributes. Each pair of items can be scored from 1 to 7 (1 represents the most negative and 7 the most positive evaluation). The items have the form of a semantic differential, i.e., each item is represented by two terms with opposite meanings. The order of the terms is randomized per item, i.e., half of the items of a scale start with the positive term and the other half of the items start with the negative term. We use a seven-stage scale to reduce the well-known central tendency bias for such types of items. In total, the questionnaire consists of six subscales. The first three dimensions are classical usability aspects, “attractiveness”, “perspicuity”, and “dependability”, whereas the last three subscales represent user experience aspects, “efficiency”, “stimulation”, and “novelty” (18). The attractiveness indicates an overall impression of the system and answers the question whether the user likes or dislikes it. The perspicuity describes whether it is easy to learn how the system works and how it is used. The dimension efficiency represents whether the product reacts fast and whether the tasks can be solved without unnecessary effort. Dependability indicates answers of the points of security and predictability, also to the control of the interaction. To have results regarding the aspects of fun, motivation, and excitement, the subscale stimulation will be analyzed. The novelty represents the design of the software in relation to creativeness and catching the interests of the users.

User experience questionnaire data belonging to the six domains were analyzed separately and not merged into a single overall score. For each item, the score was transformed from the original range (1, 7) to [−3, +3], where −3 represents the most negative and the +3 the most positive evaluation. Then, transformed score of items belonging to the same domain were averaged. For each subject, data were checked for inconsistency to detect potential random answers by the users. Since all items in a domain should measure a similar aspect of a product, we computed the difference between the best and the worst evaluation of items in a domain. In each domain, a difference higher than three was considered as an indicator of inconsistency. We decided to exclude subjects' responses that shown inconsistency for more than three domains of the scale. Median UEQ scores and interquartile ranges (IQRs) for each domain of the questionnaire were computed for each targeted disease (i.e., stroke and PD) and for the group of healthy subjects. Table 3 shows the 26 items and relative domains for UEQ.

**Table 3:** User Experience Questionnaire (18).

	**1**	**2**	**3**	**4**	**5**	**6**	**7**		**Item**	**Domain**
Annoying	o	o	o	o	o	o	o	Enjoyable	1	Attractiveness
Not understandable	o	o	o	o	o	o	o	Understandable	2	Perspicuity
Creative	o	o	o	o	o	o	o	Dull	3	Novelty
Easy to learn	o	o	o	o	o	o	o	Difficult to learn	4	Perspicuity
Valuable	o	o	o	o	o	o	o	Inferior	5	Stimulation
Boring	o	o	o	o	o	o	o	Exciting	6	Stimulation
Not interesting	o	o	o	o	o	o	o	Interesting	7	Stimulation
Unpredictable	o	o	o	o	o	o	o	Predictable	8	Dependability
Fast	o	o	o	o	o	o	o	Slow	9	Efficiency
Inventive	o	o	o	o	o	o	o	Conventional	10	Novelty
Obstructive	o	o	o	o	o	o	o	Supportive	11	Dependability
Good	o	o	o	o	o	o	o	Bad	12	Attractiveness
Complicated	o	o	o	o	o	o	o	Easy	13	Perspicuity
Unlikable	o	o	o	o	o	o	o	Pleasing	14	Attractiveness
Usual	o	o	o	o	o	o	o	Leading edge	15	Novelty
Unpleasant	o	o	o	o	o	o	o	Pleasant	16	Attractiveness
Secure	o	o	o	o	o	o	o	Not secure	17	Dependability
Motivating	o	o	o	o	o	o	o	Demotivating	18	Stimulation
Meet expectations	o	o	o	o	o	o	o	Does not meet expectations	19	Dependability
Inefficient	o	o	o	o	o	o	o	Efficient	20	Efficiency
Clear	o	o	o	o	o	o	o	Confusing	21	Perspicuity
Impractical	o	o	o	o	o	o	o	Practical	22	Efficiency
Organized	o	o	o	o	o	o	o	Cluttered	23	Efficiency
Attractive	o	o	o	o	o	o	o	Unattractive	24	Attractiveness
Friendly	o	o	o	o	o	o	o	Unfriendly	25	Attractiveness
Conservative	o	o	o	o	o	o	o	Innovative	26	Novelty

#### System Usability Scale

The SUS is a questionnaire that consists of 10 items, with five response options for each item (from “strongly disagree” to “strongly agree”), which allows the subjective evaluation of the usability of the system under the examination after the direct interaction of the user with the system (17). The administration of the usability test was aimed to understand any issues that the user encounters and consequently allow redesigning some components of the platform itself. Subjects were asked to assign a score to each item according to their level of satisfaction, after the 2 weeks of use.

System usability scale survey consists of 10 statements and each one is presented in a form of a Likert five-point scale, ranging from 1 to 5. To calculate the overall SUS score, the following formula was applied (17, 19): the item score on the positive statements was subtracted by 1 (x – 1) and the item score on the negative statements was calculated by subtracting the score from 5 (5 – x). The sum of these item scores was then multiplied by 2.5 to provide an overall SUS score between 0 (extremely poor usability) and 100 (excellent usability). SUS score above a 68 (corresponding to 50th percentile) is considered above average and the system usability acceptable. A SUS score higher than 80 (90th percentile) indicates that the system usability is excellent. Finally, the usability of the system is not acceptable when the SUS score is below 50 (33th percentile). The 10 items used for the SUS assessment are reported in Table 4.

**Table 4:** System Usability Scale (17).

	**Strongly disagree**				**Strongly agree**
I think that I would like to use this system frequently.	1	2	3	4	5
I found the system unnecessarily complex	1	2	3	4	5
I thought that the system was easy to use.	1	2	3	4	5
I think that I would need the support of a technical person to be able to use this system	1	2	3	4	5
I found that the various functions in this system were well-integrated.	1	2	3	4	5
I thought there was too much inconsistency in this system	1	2	3	4	5
I would imagine that most people would learn to use this system very quickly	1	2	3	4	5
I found the system very cumbersome to use.	1	2	3	4	5
I felt very confident using the system	1	2	3	4	5
I needed to learn a lot of things before I could get going with this system	1	2	3	4	5

#### Qualitative Semi-structured Interview

In the context of *end-user's perspective* phase, the qualitative semi-structured interview was carried out in order to collect end-users' open feedback in relation to the following four topics: (i) evaluation of affinity with technological devices, (ii) evaluation of sensor intrusiveness perception, (iii) evaluation of the rehabilitation activities provided by the vCare system, and (iv) evaluation of the VC as an ongoing service.

In the field study phase, the aim of the qualitative semi-structured interview was to gather patients' open feedback regarding the evaluation of both motor and cognitive rehabilitation experiences. Specifically, these interviews were structured following the UEQ template, which extrapolates the main idea behind the strongest items and relates them to the vCare solutions. Compared with more quantitative evaluations, this type of interview could allow users' opinion, regarding specific experiences, to be known more explicitly, perhaps making the evaluation more effective.

Qualitative semi-structured interview information, for both end-user's perspective and field study phases, was stratified according to the topics under the evaluation. The main idea from respondents was extrapolated and summarized. Table 5 shows the six questions used for guiding the semi-structured interview based on the UEQ domains.

**Table 5:** Questions of semi-structured interview extracted from the UEQ's domains.

**Domain**	**Question**
**Attractiveness**	Do you like or dislike the product?
**Perspicuity**	Is it easy for you to get familiar with the product?
**Efficiency**	Can you solve your tasks without unnecessary effort?
**Dependability**	Do you feel in control of the interaction?
**Stimulation**	In your opinion, is it exciting and motivating to use the product?
**Novelty**	Do you consider the product innovative and creative?

### Statistical Analysis

After the completion of the data collection stage, the responses to UEQ and SUS were stored in spreadsheet for further statistical tests. Boxplot and barplot were used to represent the demographic information, UEQ and SUS scores. Distributions were checked for normality with a one-sample Kolmogorov–Smirnov test.

User experience questionnaire scores are presented as median with IQR. SUS scores are presented as mean with standard deviation (SD). To test the statistical significance differences, Kruskal–Wallis H test, Mann–Whitney U test, and independent-samples *t*-test were utilized to compare UEQ data for each domain between the whole group of patients and healthy elderly. A one-way analysis of variance (ANOVA) was carried out to look for the significant differences between the two targeted diseases and healthy elderly in UEQ scores for each domain and in SUS scores. The ANOVA analysis, at this stage, was only aimed at providing additional information without any oversimplification. Furthermore, Pearson's correlation test was employed to examine the association between SUS score and the variables of demographic information. The significance of statistical tests was set at α = 0.05.

## Results

### Participants

A group of 30 patients (14 with poststroke condition and 16 with PD), 13 healthy elderly, and six health professionals (four neuropsychologists, one physiotherapist, and one bioengineer) were enrolled from two clinical centers during the two phases of participatory design (i.e., the end- user's perspective and the field study phase). All enrolled participants completed the entire proper experimental procedure.

A summary of participants' demographic characteristics divided per phase is presented in Table 6.

**Table 6:** Summary of participants' characteristics.

		**Stroke**	**PD**	**HS**	**HP**
*End-user's*	No.	6	7	13	/
*perspective phase*	Age (year) mean (std)	78.87 (9.81)	62.57 (7.57)	60.00 (12.84)	/
	Sex (M/F)	1/5	6/1	4/9	/
*Field study*	No.	8	9	/	6
*phase*	Age (year) mean (std)	74.50 (15.67)	65.56 (7.80)	/	31.75 (4.50)
	Sex (M/F)	4/4	8/1	/	2/4

*Labels in 1st row refer to the following: PD, Parkinson's disease; HS, healthy subjects; HP, healthy professionals; Labels in 2nd column refer to the following: M, male; F, female*.

Specifically, during the end-user's perspective phase, a total of 13 patients were recruited: six subjects with stroke (1/5 M/F; age 78.87 ± 9.81, years, mean ± SD) were enrolled through CCP; seven patients with PD (6/1 M/F; age 62.57 ± 7.57) were enrolled through OSA. A total of 13 healthy subjects (4/9 M/F; age 60.00 ± 12.84) were recruited from the two centers.

In the field study phase: eight patients with stroke (4/4 M/F; age 74.50 ± 15.67; 7/1 ischaemic/haemorrhagic) were enrolled from CCP; nine patients with PD (8/1 M/F; age 65.56 ± 7.80; Hoehn and Yahr scale 2.28 ± 0.62) were enrolled from OSA. Additionally, six health professionals from the two clinical centers (2/4 M/F; age 31.75 ± 4.50) were also involved.

### UEQ Scores

**Figure 2:**
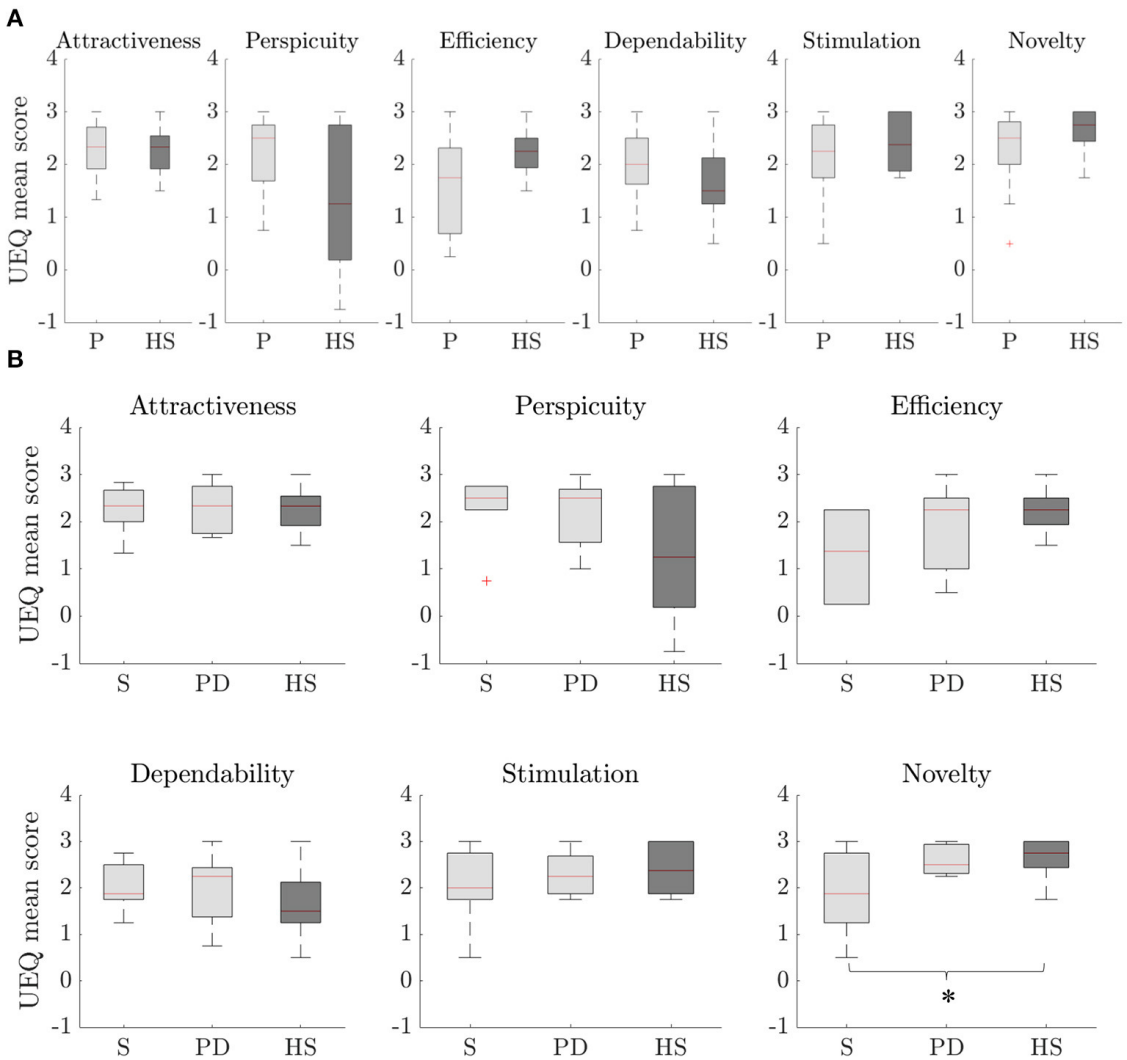
UEQ scores for each domain of the scale. Distributions are composed by the mean UEQ score in the domain for each subject. **(A)** comparison between the overall group of patients (P) and healthy subjects (HS), **(B)** comparison among the two targeted pathologies [i.e., stroke (S) and PD] and healthy subjects (HS) for each domain of the scale. Statistically significant difference with *p* < 0.05 is marked with *.

Results from UEQ are presented in Figure 2. Overall, a positive evaluation (i.e., UEQ median score > 1) was obtained for each domain of the scale in the both groups of patients and healthy subjects.

Globally, patients resulted in higher median scores than healthy subjects in half UEQ domains: attractiveness, perspicuity, and dependability. None of the domains resulted in significant differences in the comparison between the two groups (see Figure 2A).

Differentiating the groups of patients in the two targeted pathologies and healthy subjects, the one-way ANOVA resulted in a statistically significant difference only in the novelty domain of the scale (*p* = 0.038) (see Figure 2B). For this domain, *post hoc* comparisons revealed a statistically significant difference between stroke (1.86 [1.50], median [IQR]) and healthy subjects (2.75 [0.56]) (*p* = 0.031).

### SUS Scores

A total of 34 SUS questionnaires were filled out by enrolled patients; 17 SUS scales were referred to motor activities of vCare solution platform and 17 to cognitive ones.

**Figure 3:**
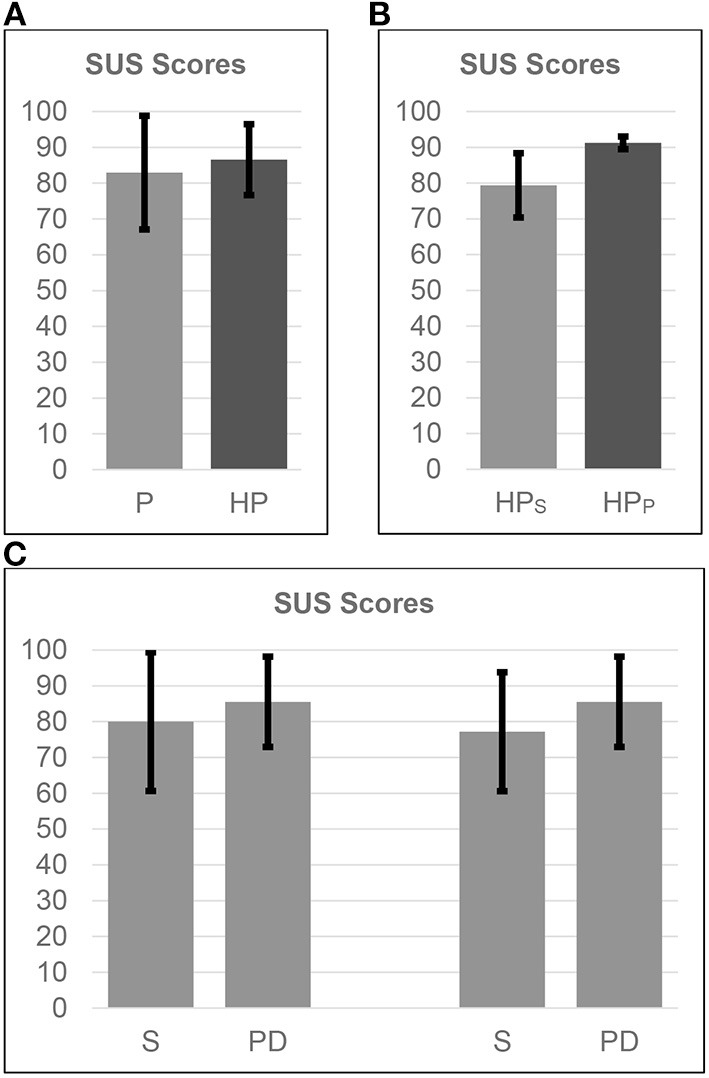
SUS scores, mean and SD. **(A)** comparison between the overall group of patients (P) and healthcare professionals (HP), **(B)** comparison among HP related to the two clinical domains [i.e., stroke (HP_S_) at CCP, PD (HP_PD_) at OSA], **(C)** comparison among the two targeted pathologies (i.e., stroke (S), PD) related to motor (first two bars) and cognitive (last two bars) activities provided by virtual coaching.

The evaluation of the overall vCare system (i.e., motor and cognitive activities) usability was rated 82.28 ± 15.12 (mean ± SD) (Figure 3). Generally, patients from the two centers assessed a comparable usability score for both motor and cognitive (82.94 ± 15.87 and 81.62 ± 14.79, respectively) rehabilitation activities (Figure 3). Concerning only the motor rehabilitation, a greater SUS score was awarded by patients with PD (85.56 ± 12.61) compared to patients with stroke (80.00 ± 19.36). Patients with PD rated the usability of the cognitive rehabilitation equal to motor one (85.56 ± 12.61), while those who suffered from a stroke concluded for a lower usability score (77.19 ± 16.61). All comparisons show a *p*-value >0.05, resulting in no statistically significant difference between groups.

The evaluation of the vCare system in terms of process of patient's characterization and rehabilitation plan's definition was evaluated as excellent from health professionals (83.33 ± 9.31). Healthcare professionals involved in caring of patients with PD showed SUS score over threshold (91.25 ± 1.77), whereas the ones with expertise in caring of patients with stroke presented a score of 79.38 ± 8.98.

Analysis between SUS scores and the patients' age showed no correlation in the overall analysis.

Moderate correlations (−0.50 < *R* < −0.75) were found only in patients with PD.

### Qualitative Semi-structured Interviews

The main considerations provided by subjects during semi-structured interviews are reported as qualitative results and organized according to the main topics assessed in the two testing phases.

#### Qualitative Semi-structured Interview Results of the End-User's Perspective Phase

For what concerns the affinity with technological devices, the majority of recruited subjects did not show particular concerns in interacting with technological tools as long as they are properly instructed and trained by skilled personnel. The main suggestion that emerged by interviewing both patients and healthy subjects was related to the software accessibility. End-users recommended to keep a low level of interaction with the device (e.g., avoid complex processes to manage, such as answering frequently through keyboard typing or button clicking). To this regard, subjects showed higher appreciation to oral conversations with the VC, although short and direct sentences are preferred to long conversational events.

In assessing the *sensor intrusiveness perception*, it emerged that sensors for the environmental and indoor monitoring integrated in vCare were not perceived as intrusive by the majority of respondents. Subjects stated that, as long as they can recognize healthcare professionals behind the technological architecture monitoring them through dedicated dashboards, they feel confident about their data protection. On the other hand, few subjects raised doubts related to privacy issues from the remote monitoring.

Regarding the *rehabilitation activities provided by the vCare system*, particular emphasis was given by subjects to the serious games for both physical and cognitive rehabilitation. From the demonstration video, subjects perceived the physical rehabilitation through serious games engaging, even if some concerns arose about safety issues for games including balance rehabilitation. Subjects showed appreciation for the possibility to monitor the indoor behavior of patients with related correctional feedback. In this regard, users highlighted the importance of receiving motivational feedback in line with the psychological status of the patient. No data protection issues were raised on these activities.

Considering VC as an ongoing service, in an overall perspective of benefits and limits of the vCare system, subjects appreciated the multiple clinical context (i.e., different diseases are considered for rehabilitation with the system), the focus of the system on QoL improvement, the multiple healthcare professionals' skills combined in a novel service, and the different rehabilitation approaches, provided seven days a week at home without excluding in-hospital examinations.

#### Qualitative Semi-structured Interview Results of the Field Study Phase

According to the majority of patients, the rehabilitation service through the *vCare* was reported to be interesting, engaging, entertaining, challenging and useful for improving impaired motor functions, and making patients aware of their cognitive abilities. Particularly for motor activities, the overall rehabilitation experience was considered to be able to stimulate movement consciousness and the real-time interaction with the screen, without using intrusive devices (e.g., controllers and wearable devices) to command the pointer, helped to enhance product attractiveness. Some patients particularly appreciated system's feedback on motor game score and the fact that having this system installed at home would mean not necessarily leaving the house for physical activity. In general, the interaction with both motor and cognitive games was deemed almost natural, easy, intuitive, and well explained, despite the fact that most patients did not have daily practicality with technology. In fact, only a few patients did not feel very much in control with the interaction because of a personal lack of technological affinity. In the specific case of cognitive games, according to most of the patients, no unnecessary efforts were required to solve the tasks although some of them were more complicated than others; tasks were almost well explained and many patients thought to be able to complete cognitive games on their own. Only in few cases, neurophysiologist's instructions were initially indispensable.

Some system bugs, in terms of sensitivity and accuracy, were also highlighted by the patients.

Definitely, most of the patients would have like to continue exercising with vCare solution at home, because they considered that the majority of serious games are exciting and motivating; some patients reported experiencing benefits and one of them declared that cognitive activities could support the traditional therapy. On the contrary, few patients believed that cognitive games are too elementary, boring, and uninspiring, which makes this activity appear less useful than more common leisure activities. In addition, the particular case of motor games seemed to be mostly indispensable in the prescription of this kind of rehabilitation by the physician, with the guarantee of improvements in patients' physical impairments. In assessing the overall solution novelty, most of the patients consider it to be innovative, although few have already had experience with similar game modality. Some other critical issues were emerged. For few patients, cognitive solution did not seem so innovative because, in their opinion, better games are available nowadays; thanks to technology and, moreover, they did not consider the usefulness of this approach to be very high for patients with their disabilities. Furthermore, not every patient thought that the cognitive solution was creative. Concerning specifically the motor games, a small number of patients did not consider the system a viable and effective substitute of the traditional physical rehabilitation; it could be only a creative, useful, and enjoyable entertaining activity. The need for a motor rehabilitation that is more tailored to the needs of patients also emerged.

## Discussion

Novel technological solutions should be developed and implemented considering the real needs and wants of the ultimate end-users. To this end, most recently, UCD approaches are encapsulated in the development stages to address end-users' real needs and avoid poor final acceptance (15). The rational under UCD is indeed that the ‘purpose of any design is to serve the user, not to use a specific technology or to be an elegant piece' [(15), p. 915, (20)].

In the case of applications in digital health for rehabilitative purposes, acceptability and usability determine a key role in the final usage of a product, especially in the elderly. The introduction of smart solutions, as VCs, has emerged more and more in the last years to provide personalized home rehabilitation programs for people in need of care. In order to make an impact in this field, the acceptance and effectiveness of such solutions is crucial (16).

The EC-funded project vCare adopted a UCD strategy from the beginning of the evaluation process. Users are included in the creation through participatory design activities meant to engage them to participate actively in the virtual coaching codesign. The main goal is to guarantee a final release fully consistent with users' expectations.

Participatory design activities that are described here were meant to engage end-users to participate actively in the virtual coaching design process (codesign process) in order to guarantee a final release fully consistent with users' expectations. The approach is meant to be incremental for a fine-tuning of the prototype: first preliminary impressions were gathered from a demonstration video (end-user's perspective phase), and then the transition to a field study allowed to test the usability of the system coming from direct experience in a controlled environment and the distance between projection and direct use.

### User Perception of vCare Solution

Results from end*-*user's perspective phase showed globally a positive preliminary perception of the service. All domains of the UEQ scale showed similar evaluations of the system from both the patients' perspective and the healthy subjects' perspective. Evaluations from the two groups seem to suggest that, even if the system is addressed to pathological subjects, it is similarly perceived as attractive, interesting, and innovative from healthy people. Subgroups investigation showed consistent results with the patients vs. control evaluation, except for the novelty domain of the scale. Differences in ratings of this domain may be explained by the formulation of the related UEQ items linked to the absence of a direct experience of the system at this stage. Indeed, novelty is measured in terms of how the user retains captivating and engaging the software design. Since subjects were not directly using the system, they could have had a different perception of these aspects.

From these first results correlated with qualitative feedback from the open discussions, some important remarks emerged associated with the user-friendliness of the system. “Attention points” that should be taken into account in refining the solution are related mainly to an easy-to-use interface and software accessibility by elderly people. In such technological solutions, the interaction of the patient with the system should be natural and prompt in order to limit the abandonment of the use. Moreover, strong identification of healthcare professionals operating behind the system is crucial. As long as patients can feel the assistance and follow-up from a real healthcare professional to the rear of the technological infrastructure, sensor intrusiveness is perceived as minimal. To this purpose, specific dashboards for medical doctors, physiotherapists, and neuropsychologists are being integrated in vCare in order to monitor patient's needs and improvements. Finally, although a VC cannot provide the empathy of a human caregiver, the ‘psychological' dimension (e.g., correctional feedback and positive stimuli) should be well-finished to empower the patient to pursue correct habits, similarly to a real career.

### Usage and Acceptability of Proposed Solution

After a first presentation of vCare through demonstration videos, during the field study phase, a group of patients tested the vCare system for the first time in a ‘controlled environment'.

Overall, results from SUS questionnaires were revealed above the threshold reported in the literature to define an excellent usability of the system (SUS score > 80), in both patients and health professionals. None of the subgroups of patients rated the system with a poor usability; lower SUS scores were assigned by stroke survivors, more markedly for cognitive rehabilitation. However, no statistically significant difference in the SUS score was found between groups.

Lower SUS score regarding the cognitive activities, while remaining generally good, probably reflects the more critical issues emerged from the semi-structured interview evaluation for the cognitive solution; system bugs, as problems with tablet's touch sensitivity and accuracy, contributed to worse patients' feedbacks together with the need of a greater variability and difficulty of games.

Overall, the rehabilitation experience was considered as positive and useful, an interesting way to practice physical activity at home. Indeed, the interview revealed that some patients with PD considered that it is useful to have the vCare rehabilitation system at home to achieve a better follow-up of disease's symptoms and prevent the unavoidable decline due to social distancing required by the COVID-19 pandemic period.

The high usability values obtained could be corroborated also by the intuitiveness of the system interaction; progressive confidence with this technology was referred by most of the patients in subsequent sessions, allowing an independent use of it at home without many troubles. Indeed, most of them were confident that they would be able to interact with the system autonomously without further overview, due to the almost ease of use and right level of accessibility with respect to their technological attitude. Only in few cases, further instructions and support were considered necessary; user manuals and/or short demo videos to remind how to use the system would have been appreciated. This result, in particular, seems to satisfy the requirements that emerged from the end-user's perspective phase: easy-to-use interface and natural interaction with the system. Therefore, although not for everyone, this solution could effectively replace conventional therapy, most patients would have been interested in continuing this innovative vCare rehabilitation program at home.

Finally, results obtained from the interviews could also explain the reason why motor activity's usability values that close to the maximum level were not achieved. Indeed, patients remarked that some improvements in the system responses and in the accuracy of movement recognition were needed in order to avoid frustration. Moreover, the necessity emerged for the system to be more customized and related to patients' needs; sometimes, a more immersive and realistic game environment with simulation of real-life scenarios and a higher complexity level of the games would have been appreciated in order to stimulate a more constant physical activity. In fact, although some exercises were declared to be more complicated than others, requiring a good level of attention and a positive attitude especially using the impaired limbs, one patient considered that motor games are not excessively tiring.

### Limitations

One of the limitations of the work is the low number of the users, however justified by the preliminary and explorative aspect of this investigation. Despite the quantitative analysis proved interesting, due to low number of involved users, the statistical analysis, at this stage, was only aimed at providing additional information without any oversimplification. For this reason, in the discussion section, we have avoided any inference or generalization of the reported results.

In fact, a further phase (Pilot Tests Phase) has already been foreseen. On the other hand, we observed a positive patient's attitude in collaborating in the development of a coaching system of which he/she will become a user.

During both phases of this work, the collection of qualitative data (semi-structured interviews) was privileged with the aim of maintaining constant that the dialogue with patients and not reducing their participation to a simple parametric evaluation. Another limit is the poor evaluability of the progression of the patient's participation from the speculative analysis of the solution (i.e., informational material and video footage) (*end-user Perspective* phase) to the examination of the proposed “product” (i.e., the vCare solution) (*field study* phase). As strategy to mitigate this risk, both semi-structured interviews that proposed in the two phases were created following UEQ template.

### Future Studies

The last and longest phase of validation will be performed in the near future after refining the system according to the feedback gathered from users in the first two phases.

This phase will be the last step of clinical validation of the system. It is planned as a randomized controlled trial (RCT) where the implemented and tested system will be clinically validated at users' homes to measure the impact of the new vCare rehabilitation coaching service. Groups of patients will be enrolled for each clinical site according to specific enrolment criteria and will follow a personalized rehabilitation program with vCare at home during a 6-month period. Acceptability of the solution, clinical improvement, risk reduction, and overall QoL improvement will be deeply investigated and evaluated. Further details about this last phase of clinical validation are presented in Kyriazakos et al. (3).

Future efforts need to greatly extend the demographic, socioeconomic, and cultural reach of the populations addressed, to test whether levels of acceptability are maintained, and if not, how these can be achieved. Finally, future works should incorporate new evidence-based interventions and incorporate them into the platform.

## Conclusion

The continuity of care in chronic diseases or after acute episodes is often interrupted once the patient has been discharged from the hospital. To fill this gap, the EC-funded project “vCare” (Virtual Coaching Activities for Rehabilitation in Elderly) aims to develop a smart solution based on a VC providing personalized home rehabilitation programs for patients affected by stroke, Parkinson's disease, heart failure, and ischemic heart disease. The project incorporates the involvement of final users in each stage of development to create a solution compliant to end-users' needs and preferences.

To the current stage of system development, user experience and usability of the platform were tested in a two-fold assessment. Results indicated good ratings of the product and, in general, high level of enthusiasm and curiosity by neurological patients. Indeed, several of them would be glad to use vCare system at home. Moreover, suggestions about possible improvements were gathered from open feedback and discussions.

Our result seems to satisfy an easy-to-use interface and an intuitive interaction between users and the developed platform. Consequently, although with some limitations, the proposed solution could be, in the next future, a valid rehabilitative program at home together with standard treatments.

Once refined and fine-tuned in the aspects highlighted in the present evaluations, the system will be clinically tested at user's home to measure the real impact of the rehabilitative coaching services.

As a matter of fact, users' feedback gathered during the testing phases will be taken into consideration along the development and refinement process in terms of functional optimisation, customization, and further patient's engagement to increase the final acceptance and the vCare use adherence.

## Data Availability Statement

The datasets recorded and/or analysed during the current study are available from the corresponding author on reasonable request.

## Ethics Statement

The studies involving human participants were reviewed and approved by Comitato Etico Milano Area 2. The patients/participants provided their written informed consent to participate in this study.

## Author Contributions

AS, ET, and PT analyzed the data, interpreted the results, and took the lead in writing the manuscript. AS and ET contributed equally to the work. All authors provided critical feedback and helped to shape the manuscript.

## Funding

This work was partially supported by the EC within the vCare project (funded by Horizon 2020 Research and Innovation Programme under the Grant Agreement No. 769807).

## Conflict of Interest

The authors declare that the research was conducted in the absence of any commercial or financial relationships that could be construed as a potential conflict of interest.

## Publisher's Note

All claims expressed in this article are solely those of the authors and do not necessarily represent those of their affiliated organizations, or those of the publisher, the editors and the reviewers. Any product that may be evaluated in this article, or claim that may be made by its manufacturer, is not guaranteed or endorsed by the publisher.
